# Association of Suicide Attempt with Stimulant Abuse in California Emergency Departments in 2011: A Study of 10 Million ED Visits

**DOI:** 10.5811/westjem.2021.8.51022

**Published:** 2022-02-23

**Authors:** Shahram Lotfipour, Nikhil Shah, Hina Patel, Soheil Saadat, Tim Bruckner, Parvati Singh, Bharath Chakravarthy

**Affiliations:** * UC Irvine School of Medicine, Department of Emergency Medicine, Irvine, California; † Eisenhower Health, Department of Emergency Medicine, Rancho Mirage, California; ‡ Feinberg School of Medicine-Northwestern University, Chicago, Illinois; § UC Irvine School of Biological Sciences, Irvine, California; ¶ UC Irvine Program in Public Health, Irvine, California; || UC Irvine School of Medicine, Department of Public Health, Irvine, California

## Abstract

**Introduction:**

Our goal in this study was to identify stimulant abuser patients who are at specifically high risk of suicide attempt (SAT), in order to prioritize them in preventive and risk mitigation programs.

**Methods:**

We used the California State Emergency Department Database (SEDD) to obtain discharge information for 2011. The SEDD contains discharge information on all outpatient ED encounters, including uninsured patients and those covered by Medicare, Medicaid, and private insurance. We identified SAT and stimulant abuse by using the relevant International Classification of Diseases, Ninth Revision, codes.

**Results:**

The study included 10,124,598 outpatient ED visits. Stimulant abuse was observed in 0.97% of ED visits. Stimulant abuse was more common among young and middle-aged males and people with low median household income. Moreover, it was more common among Native American (1.8%) and Black (1.8%), followed by non-Hispanic White (1.1%) patients. The prevalence of SAT was 2.0% (N = 2000) for ED visits by patients with a history of stimulant abuse, and 0.3% (N = 28,606) for ED visits without a history of stimulant abuse (odds ratio 7.29, 95% confidence interval, 6.97–7.64). The SATs were directly associated with stimulant abuse, younger age (age groups >10), and non-Hispanic White and Native American race. Association of SAT with stimulant abuse was stronger in female patients.

**Conclusion:**

Stimulant abuse was the only modifiable risk factor for suicide attempt in our study. Reaching out to populations with higher prevalence of stimulant abuse (young and middle-aged individuals who are Native American or Black, with lower household income) to control the stimulant abuse problem, may reduce the risk of SAT. In this regard, people who are at higher risk of SAT due to non-modifiable risk factors (younger age, and Native American or White race) should be prioritized. Moreover, controlling stimulant abuse among women may be specifically effective in SAT prevention.

## INTRODUCTION

The use and abuse of stimulants has been increasing across the United States.^1^ This includes rates of recreational cocaine use along with medical and nonmedical amphetamine consumption. The risk, severity, and type of stimulant abuse have been shown to vary across different populations.^1^ Various trends have been established in regard to different population demographics. For example, it has been shown that methampheta-mine is more prevalent in the western US, although this has been trending eastward.^2^

Cocaine and amphetamines have different mechanisms of action but similarly affect monoamine transporters. Cocaine blocks the reuptake of neurotransmitters, while amphetamine releases more into the synapse.^2^ Therefore, when comparing the two drugs, methamphetamine affects dopamine balance in the brain for a longer period of time. This is one of the many factors that have led to the differential effects of these stimulants.^3^

In recent years, there has been an increase in overall prescriptions to college students, especially to those in academically stressful situations.^4^ Misuse of stimulants has been shown to cause multiple issues including tissue ischemia and long-term neurological changes. An apparent correlation has been observed between the increase in overall stimulant prescription to patients of varying ages and demographics and misuse of these stimulants, resulting in both physiological and neurological changes that could be prevented.^5^ Particularly, the neurological changes that result from stimulant abuse may increase their risk of suicide. Globally, suicide is the third leading cause of death in the 15–44 age group.^6^ Although a strong correlation between stimulant abuse-induced neurological changes and suicide exists, various other factors contribute to the onset of suicidal thoughts. The rising concern with regard to impulsive suicidal thoughts, and their potential to claim lives, has spurred public health intervention efforts to provide support to these most vulnerable and at-risk populations. Public health interventions in populations suffering from stimulant abuse can facilitate a reduction of suicide attempts (SAT) in this demographic.^5^ Specific, targeted preventive efforts may reduce SAT in at-risk populations and help maintain mental and physiological health.

An association between stimulant abuse and SAT has already been reported.^7^ We expanded on this work, accessing the 2011 State Emergency Department Database (SEDD) to determine which subgroups, if any, of stimulant-abuse populations are at increased risk of SAT. This subgroup analysis may inform targeted public health efforts focused on the most at-risk individuals.

## METHODS

We used data from the California State Emergency Department Database (SEDD) 2011 for analysis. We considered emergency department (ED) visits identified by E95* *International Classification of Diseases, Ninth Revision* (ICD-9), code as a SAT case. The visit was classified as stimulant abuse-related, if at least one of the following ICD-9 codes was associated with the visit: 304.2* (Cocaine dependence); 304.3* (Cannabis dependence); 304.4* (Amphetamine type and other psychostimulants dependence); 305.2* (Nondependent abuse of drugs: cannabis); 305.6* (Nondependent abuse of drugs: cocaine type); 305.7* (Nondependent abuse of drugs: amphetamine type); 969.7* (Poisoning by psychostimulants); 970.0 (Poisoning by analeptics); 970.8* (Poisoning by other central nervous system stimulants); 970.9 Poisoning by unspecified central nervous system stimulants); E854.2 (Accidental poisoning by other psychotropic agents: psychostimulants); E854.3 (Accidental poisoning by other psychotropic agents: central nervous system stimulants); E939.7 (Drugs, medicaments, and biological substances causing adverse effects in therapeutic use: psychotropic agents, psychostimulants) ; E940.0 (Drugs, medicaments, and biological substances causing adverse effects in therapeutic use: central nervous system stimulants, analeptics); E940.8 (Other specified central nervous system stimulants causing adverse effects in therapeutic use); and E940.9 (Unspecified central nervous system stimulant causing adverse effects in therapeutic use).

**Population Health Research Capsule**

What do we already know about this issue?

*Different populations in the US are increasingly susceptible to the use of stimulants. A strong correlation exists between stimulant abuse and suicide attempt (SAT).*

What was the research question?

*We investigated which subgroups of stimulant abuse patients in California emergency departments had higher risk of SAT.*

What was the major finding of the study?

*A SAT was associated with stimulant abuse, younger age, and being non-Hispanic White or Native American. The association was stronger in females.*

How does this improve population health?

*Suicide prevention interventions in healthcare settings should actively target patients (especially females) who have stimulant abuse issues.*

We used Stata 14.2 SE statistical software (StataCorp LLC, College Station, TX) for data analysis. Prevalence proportions are reported as percentage and 95% confidence intervals (CI). Logistic regression analysis was used to examine the association of SAT with age groups, gender, stimulant abuse, and race. Some patients had been referred more than once to the ED; therefore, the dataset was considered as clustered at the level of the patient, and the standard errors were estimated by clustered robust method.

## RESULTS

The study included 10,124,598 ED visits in California in 2011. Stimulant abuse was associated with 97,834 (0.97%; 0.96% – 0.97%) ED visits. Table 1 shows the prevalence of stimulant abuse in different patient groups. Stimulant abuse was more common among patients of young and middle age, male (1.40%), Black (1.8%), and Native American (1.8%), followed by non-Hispanic White (1.1%) patients. Stimulant abuse was more common in patients with lower household income. The prevalence of SAT was 2.0% (N = 2000) for ED visits with a history of stimulant abuse, and 0.3% (N = 28,606) for ED visits without a history of stimulant abuse (odds ratio [OR] 7.29, 95% CI, 6.97–7.64). In the state of California, 30,606 (0.30%) ED visits were associated with SAT.

### Univariable Analysis

**Figure 1:**
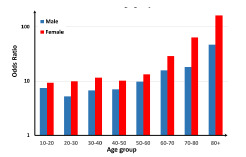
Association of suicide attempt with stimulant abuse in age-gender groups.

**Figure 2:**
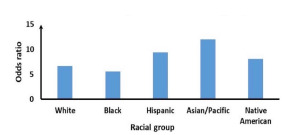
Association of suicide attempt with stimulant abuse in racial groups.

The association of SAT with stimulant abuse was stronger in women (OR 9.18, 95% CI, 8.60–9.80) compared to men (OR 6.45, 95% CI, 6.04–6.88). This pattern was seen in all age groups >10. The association of SAT with stimulant abuse was stronger in ages above 60 (OR 12.55, 95% CI, 8.68–18.16) compared to younger age groups (OR 6.43, 95% CI, 6.14–6.74). The pattern was similar in both genders (Figure 1). The association of SAT with stimulant abuse in Asian/Pacific (OR 12.01, 8.88 – 16.26) and Hispanic patients (OR 9.41, 8.59 – 10.32) was stronger than White (OR 6.66, 6.26 – 7.09) and Black (OR 5.61, 4.93 – 6.39) patients (Figure 2).

### Multivariable Analysis

In a multivariable analysis (Table 2), SAT was directly associated with stimulant abuse (OR 4.18), young age (>10), and female gender. Suicide attempts were more frequent among White and Native American patients, compared to Black. The association of SAT with stimulant abuse was stronger in female patients in multivariable analysis.

## DISCUSSION

Analysis of over 10 million ED visits in California gave us insight into the relation between SAT and stimulant abuse in different patient populations. Our findings cohere with previous findings and indicate that depressed or suicidal individuals are more likely to abuse stimulants and are increasingly susceptible to SAT. As the only modifiable risk factor in our study, stimulant abuse was more common in young and middle-aged, male, Native American, and Black patients with lower household income. We also found that stimulant abuse puts females at higher risk of SAT.

The risk of SAT is prevalent across patient populations and increases with factors such as stimulant abuse.^5^ Not only does a SAT endanger the life of a vulnerable individual, it also psychologically affects the individual, families, communities, and society as a whole. The substantial impact that suicide has on the community necessitates public health intervention efforts to target high-risk populations. Young populations have been deemed increasingly at risk of suicide due to a variety of psychosocial stressors.^5^ Research stipulates that within these diverse, young populations, females have proven to be the most vulnerable group.^5^ Suicide remains the second leading cause of death in individuals between the ages of 10–34.^6^ Stimulant abuse contributes to the numerous stressors that young populations face.^8^ Public health prevention efforts within this demographic group may reduce the economic and human cost of suicide.

The rising national trend in nonmedical prescription stimulant abuse has allowed experts to discern the psychological factors that contribute to the start of recreational substance consumption.^9^ This work indicates that the initiation of abuse often follows discrete traumatic events.^10^ Therefore, the inefficiency of prescription medication as a coping mechanism may be attributed to these higher suicidal rates. A prominent correlation between lower median household income state quartile (MHIQ) and increased stimulant abuse (MHIQ = 1.2%) exists (Table 1). Poor access to healthcare and high rates of depression in individuals of lower socioeconomic status contribute to psychological effects prompting nonmedical stimulant abuse.^8^ Non-medical stimulant use has also been associated with other harmful habits including tobacco, alcohol, and other illicit drug use.^4^ Each of these habits has also been correlated to increased suicide risk, all of which may be contributing factors.^11^

Multivariable analysis showed SAT is associated with stimulant abuse and younger age. One potential reason for this result may involve the absence of impulse control correlated with drug abuse.^12^ Meanwhile, the proportion of ED visits with associated stimulant abuse was higher in younger age groups. This pattern corroborates past research indicating increased non-medical stimulant use among college populations.^4^ Association of SAT with stimulant abuse (besides younger age), and higher prevalence of stimulant abuse in those who are younger in age indicates that young people should be targeted for active stimulant-abuse prevention and treatment interventions.

We found a stronger association between SAT and stimulant abuse in females, in all age groups. Previous literature coheres with this finding. Gender differences in stimulants have been established both behaviorally and pharmacologically. Women have been known to undergo the telescoping effect, which stipulates that in the long term, females escalate from low-dose use to addiction faster than men.^13^ The quicker increase in consumption rates has been attributed to hormonal fluctuations inherent with the menstrual cycle. This hormonal fluctuation has been shown to subject women to differential drug effects dependent on their menstrual phase.^14^ Women have been shown to be significantly more susceptible to physiological dependence, which is the most extreme classification of drug use in the *Diagnostic and Statistical Manual of Mental Disorders, Fourth Edition*. This is associated with an increase in extreme lifestyle changes attributable to drug administration and consumption.^3^

Suicide attempts are associated with Native American and White race. At the same time, stimulant abuse was more common in Native American, and to a lesser extent, White patients. This pattern indicates active stimulant-abuse prevention and treatment interventions could specifically reduce SAT in those racial groups. It has been well established in the literature that race plays a significant role in the type of substance being abused.^15^ The increased rate of cocaine abuse in Black populations has been attributed to distribution networks and the historic, structurally driven prevalence of cocaine in Black communities.^15^ White and Hispanic populations, on the other hand, have more commonly used amphetamines or are considered dual users of both stimulants.^3^ Interestingly, Asian/Pacific Islanders have also experienced a sharp increase in non-cocaine stimulant admissions to treatment centers.^16^ We were not able to differentiate the exact type of the stimulant in this study.

## LIMITATIONS

It should be noted that this study does have its limitations. First, a substantial number of patients diagnosed in the ED could have had suicidal thoughts without SAT. Additionally, patients who have intended self-harm without SAT could have been mischaracterized as SAT. This categorizes individuals who had suicidal intentions but no SAT in the same category as those who were suicidal with SAT.

## CONCLUSION

Suicide attempts were associated with stimulant abuse, younger age, and White or Native American race. Stimulant abuse was the only modifiable risk factor for SAT in our study. Therefore, we recommend that groups with higher prevalence of stimulant abuse (young and middle aged, Native American and Black race, with lower household income) be targeted for stimulant-abuse prevention and treatment to reduce SAT. In this regard, people who are at higher risk of SAT due to non-modifiable risk factors (younger age, Native American or White race) should be prioritized. Moreover, controlling stimulant abuse among women would be specifically effective in SAT prevention. The findings presented could be of value when developing screening tools to implement in a patient care setting that stratifies patients into risk categories for SAT.

## Figures and Tables

**Table 1:** Prevalence of stimulant abuse per emergency department visits, according to patients’ characteristics.

Demographics	Stimulants Abuse			

	No		Yes	
	
	Count	Row %	Count	Row %
Age group				
0–10	1,768,307	100.0%	237	0.0%
10–20	1,112,735	98.7%	14,267	1.3%
20–30	1,637,599	98.2%	29,655	1.8%
30–40	1,300,234	98.5%	19,899	1.5%
40–50	1,289,198	98.6%	18469	1.4%
50–60	1,141,177	99.0%	11,613	1.0%
60–70	712,538	99.6%	2,925	0.4%
70–80	491,645	99.9%	403	0.1%
80–90	515,341	100.0%	90	0.0%
Gender				
Male	4,465,778	98.6%	61,998	1.4%
Female	5,426,236	99.4%	35,214	0.6%
Race				
White	4,119,855	98.9%	44,413	1.1%
Black	1,079,244	98.2%	19,593	1.8%
Hispanic	3,515,411	99.3%	25,526	0.7%
Asian/Pacific	453,282	99.6%	1,799	0.4%
Native American	18,253	98.2%	335	1.8%
Other	321,250	99.1%	2,782	0.9%
Median household income state quartile for patient ZIP Code				
1	3,087,808	98.8%	38,239	1.2%
2	2,684,738	99.1%	24,087	0.9%
3	2,289,600	99.2%	18,698	0.8%
4	1,702,577	99.3%	11,243	0.7%

**Table 2:** Association of suicide attempt with stimulant abuse and different demographic characteristics.

Variable	Odds ratio	Standard error	Z	P	95% Confidence interval
Stimulant abuse					
Yes (vs No)	4.18	.159	37.67	<0.001	3.88 – 4.51
Gender					
Female (vs male)	1.07	.017	4.59	<0.001	1.04 – 1.11
Age groups					
10–20 (vs 0–10)	92.30	14.160	29.50	<0.001	68.33 – 124.68
20–30 (vs 0–10)	59.45	9.11	26.67	<0.001	44.04 – 80.27
30–40 (vs 0–10)	46.36	7.12	24.99	<0.001	34.31 – 62.64
40–50 (vs 0–10)	41.46	6.37	24.25	<0.001	30.68 – 56.02
50–60 (vs 0–10)	29.70	4.57	22.02	<0.001	21.96 – 40.16
60+ (vs 0–10)	7.89	1.23	13.25	<0.001	5.82 – 10.72
Race					
White (vs Black)	1.89	.049	24.77	<0.001	1.80 – 1.99
Hispanic (vs Black)	1.07	.030	2.43	0.015	1.01 – 1.13
Asian/Pacific (vs Black)	1.18	.054	3.55	<0.001	1.08 – 1.29
Native American (vs Black)	1.61	.228	3.35	0.001	1.22 – 2.12
Others (vs Black)	1.58	.068	10.60	<0.001	1.45 – 1.72
Interactions					
Stimulant by Female	1.49	.080	7.36	<0.001	1.34 – 1.65
Model Constant	.0001	0.00001	−63.79		

Pseudo R_2_ = 0.05 (Standard error adjusted for 4,528,235 clusters).
